# Split Course Hyperfractionated Accelerated Radio-Chemotherapy (SCHARC) for patients with advanced head and neck cancer: Influence of protocol deviations and hemoglobin on overall survival, a retrospective analysis

**DOI:** 10.1186/1471-2407-6-279

**Published:** 2006-12-07

**Authors:** Peter Stadler, Kurt Putnik, Thore Kreimeyer, Lisa D Sprague, Oliver Koelbl, Christof Schäfer

**Affiliations:** 1 Department of Radiotherapy and Radiation Oncology, University Hospital Regensburg, Germany; 2 Department of Radiotherapy and Radiation Oncology, University Hospital TU Munich, Germany; 3 Praxis Muehleninsel Landshut-Muehldorf-Dingolfing, Germany

## Abstract

**Background:**

The advantage of hyperfractionated accelerated radiation therapy for advanced head and neck cancer has been reported. Furthermore, randomized trials and meta-analyses have confirmed the survival benefit of additional chemotherapy to radiotherapy. We retrospectively analyzed the efficiency and toxicity of the Regensburg standard therapy protocol "SCHARC" and the overall survival of our patients.

**Methods:**

From 1997 to 2004, 64 patients suffering from advanced head and neck cancer (88 % stage IV, 12 % stage III) were assigned to receive the SCHARC protocol. Around half of the patients were diagnosed with oro-hypopharynx carcinoma (52 %), one third with tongue and floor of mouth tumors (29 %) and one fifth (19 %) suffered from H & N cancer at other sites. The schedule consisted of one therapy block with 30 Gy in 20 fractions over a two week period with concomitant chemotherapy (d 1–5: 20 mg/m^2^/d DDP + 750–1000 mg/m^2^/d 5FU (cont. infusion). This therapy block was repeated after a fortnight break up to a cumulative dose of 60 Gy and followed by a boost up to 70 Gy (69–70.5 Gy). All patients assigned to this scheme were included in the survival evaluation.

**Results:**

Forty patients (63 %) received both radiation and chemotherapy according to the protocol. The mean follow up was 2.3 years (829 d) and the median follow up was 1.9 years (678 d), respectively. The analysis of survival revealed an estimated 3 year overall survival rate of 57 %. No patient died of complications, 52 patients (80 %) had acute grade 2–3 mucositis, and 33 patients (58 %) suffered from acute grade 3 skin toxicity. Leucopenia was no major problem (mean nadir 3.4 g/nl, no patient < 1.0 g/nl) and the mean hemoglobin value decreased from 13.2 to 10.5 g/dl. Univariate analysis of survival showed a better outcome for patients with a hemoglobin nadir >10.5 g/dl and for patients who completed the protocol.

**Conclusion:**

The SCHARC protocol was effective in patients diagnosed with advanced head and neck cancer. It led to long-term disease control and survival in about 50 % of the patients with significant but acceptable toxicity. Most patients were not anemic at beginning of therapy. Therefore, we could assess the influence of pre-treatment hemoglobin on survival. However, a low hemoglobin nadir was associated with poor outcome. This result suggests an influence of anemia during therapy on prognosis.

## Background

The optimal treatment for head and neck cancer is still a matter of dispute. In resectable tumors, complete resection followed by adjuvant radio- or radiochemotherapy revealed excellent local control rates but survival is impaired by second malignancies and distant metastases [1]. However, in advanced, non resectable head and neck cancer, local tumor control still predicts the outcome. Therefore, many approaches have aimed at reducing the rate of local treatment failures [2].

### Rationale of the SCHARC treatment regimen

The application of hyperfractionated accelerated radiation therapy for advanced head and neck cancer has been shown to be of major advantage in comparison to normofractionated schedules or accelerated radiation therapy alone [3]. In addition, randomized trials and meta-analyses have confirmed the survival benefit of additional chemotherapy to radiotherapy. Moreover, some authors recommend radiochemotherapy followed by surgical resection of the reduced tumor masses [4-6].

Split course radiotherapy in standard fractionation schemes is known to be disadvantageous for patients with head and neck cancer. However, the detrimental effect of a pause can be compensated by accelerated hyperfractionation [3]. Ernst-Stecken et al. recently reported encouraging results in a phase II study on hyperfractionated accelerated split course radiochemotherapy in head and neck cancer [7]. This study revealed a remarkable local control rate of 78 % and an overall survival rate of 49 % after a 3 year observation period. A comparable treatment regimen was also established as the Regensburg standard protocol "SCHARC" from 1997 to 2004.

### Prognostic significance of hemoglobin level of cancer patients

Recently, strong evidence has been found concerning the role of anemia and tumor oxygenation as prognostic factors [8-12]. Some studies with hypoxic cell sensitizers or tumor oxygenation modification protocols reported an improved survival rate [13,14]. Anemia is the most important cause of tumor hypoxia. Therefore, it is not surprising that anemia can influence the prognosis of cancer patients. Anemia during therapy (hemoglobin nadir) is worth to be considered as a prognostic factor because it frequently is a consequence of the therapy modality (surgery, chemotherapy). Correcting anemia with transfusions and application of erythropoietin therefore seems to be a valid approach to compensate for the negative effects of anemia. However, in a current randomized trial correction of anemia with erythropoietin did not result in survival benefit. Hence, erythropoietin should be administered with care and only within a study.

We retrospectively analyzed the local efficiency and toxicity of the Regensburg standard therapy protocol "SCHARC" and the overall survival of our patients. This protocol was not a study protocol but a protocol for patients not encompassed in any study whatsoever. Additionally, we investigated the role of protocol deviations and anemia as potentially relevant factors for the overall survival.

## Methods

From 1997 to 2004, 64 patients suffering from advanced head and neck cancer (88 % stage IV, 12 % stage III) were assigned to receive the SCHARC protocol at the Department of Radiation Oncology at the University of Regensburg. All patients had been considered as inoperable by a board of ENT-surgeons and radiation oncologists and were without any contraindications against the treatment scheme. The patients had been considered as inoperable due to tumor location, size, and extension or because of comorbidities. The patients were on average 56 (± 8) years old and mostly of male gender (see Table 1). Most patients (58 %) had a history of combined alcohol and nicotine abuse. 9 % were non smokers, 17 % had no alcohol abuse in their anamnesis and 3 % of the patients had neither a history of alcohol nor nicotine abuse. About half of the patients were diagnosed with oro-hypopharynx carcinoma (52 %), one third with tongue and floor of mouth tumors (29 %) and one fifth (19 %) suffered from head and neck cancer at other sites (Table 1).

The staging included clinical examination, CT or MRI scan, ultrasound of the neck and upper endoscopy by the head and neck surgeon with biopsies of the tumor regions.

66 % of the tumors were classified as T4. 24 % and 10 % were categorized as T3 and T2 tumors, respectively. 15 % were staged as N3 and 65 % were classified as N2. Only 9 % and 11 % were categorized as N1 and N0 (Table 1). According to the UICC (1992) classification 88 % of the patients were stage IV and 12 % stage III.

Prior to start of therapy a percutaneous endoscopically controlled gastrostomy (PEG) was given to all patients. All patients gave informed consent to the treatment.

### Radio- and Chemotherapy

The PTV (planning target volume) included the primary tumor region and the regional lymphatic drainage of the neck. All patients were planned with a computer aided 3D-planning system (different versions (4.0–6.1) of the Helax TMS™). The common technique were two lateral isocentric opposed portals for the cranial part of the PTV combined with an anterior or two anterior/posterior opposed portals for the caudal part of the PTV (including a midline absorber if reasonable). After 39 Gy the spinal cord was shielded and the posterior neck nodes were treated with 9–12 MeV electron beam up to 60 Gy. The radiotherapy was given with 6 MeV photons by a Siemens Linear Accelerator (Primus) with MLC's.

The schedule consisted of one therapy block with 30 Gy in 1.5 Gy twice a day over a two week period with concomitant chemotherapy (d 1–5: 20 mg/m^2^/d DDP + 750–1000 mg/m^2^/d 5FU (cont. infusion)). This therapy block was repeated after a fortnight break up to a cumulative dose of 60 Gy and followed by a boost up to 70 Gy (69–70.5 Gy).

### Assessment of Toxicity

The acute toxicity was usually classified according to the CTC criteria [16]. Patients were seen once a week and skin and mucosa toxicities were documented in the chart. In 5 % of patients the grade of mucosal toxicity was not documented according to CTC criteria. At least once a week complete blood cell count and kidney associated parameters (creatinin, electrolytes) were checked. Late toxicity was classified according to the LENT-SOMA scale [17].

### Definition of protocol violation

Application of Mitomycin C (n = 4) and Carboplatin (n = 4) instead of DDP (due to limited renal function) was considered a protocol violation. No or incomplete hyperfractionation (n = 11) due to skin toxicities or strongly deteriorated general condition was also considered a major protocol deviation. 4 patients discontinued the therapy. The additional application of folic acid (n = 4) was not considered as a protocol violation (minor deviation).

### Statistics

Overall survival was defined as the time interval from the start of radiochemotherapy to either the date of death (event) or to the date of last contact with the patient. Freedom from local treatment failure (FLTF) was defined as the time interval from radiochemotherapy to either the date of recurrence (event) or to the date of last contact with the patient or to the date of death. First follow-up examination was done by the head and neck surgeons and the radiation oncologists 6 weeks after ending of therapy. The standard procedure of the first follow up examination included CT or MRI scan, ultrasound of the neck and upper endoscopy with biopsy of suspicious areas. The head and neck surgeon examined the patients every three months by means of upper endoscopy and ultrasound of the neck. These intervals were increased to six months after two years of follow-up. Radiotherapy follow-up was performed twice a year in the first two years and once a year after the second year.

Patients were grouped by pre-therapy hemoglobin value and hemoglobin nadir to assess for the influence of hemoglobin on the survival probability. We used the median pre-treatment hemoglobin level of 12.5 g/dl as the cut off value for the pre-treatment analysis and the median of hemoglobin nadir of 10.5 g/dl as the cut off point for the analysis during treatment. The median hemoglobin values were used as cut off points to obtain two approximately equal sized patient groups.

The possible influence of other factors leading to a drop in hemoglobin values, such as weight loss or deteriorated general condition was not analysed and can therefore not be ruled out. Overall survival and FLTF probability were estimated by means of Kaplan Meier analysis using the Cox-proportional hazards regression model. We used the commercially available WinSTAT^® ^for Microsoft^® ^Excel Version 1999.2 software for data analysis.

## Results

### Outcome

**Figure 1:**
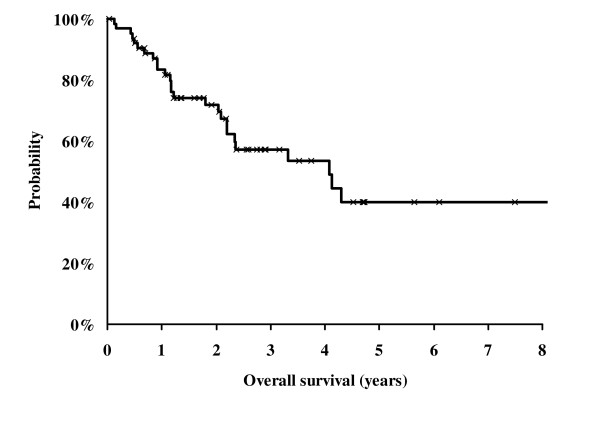
Kaplan Meier Analysis of survival probability of all 64 patients assigned for the SCHARC-scheme (intention to treat analysis).

**Figure 2:**
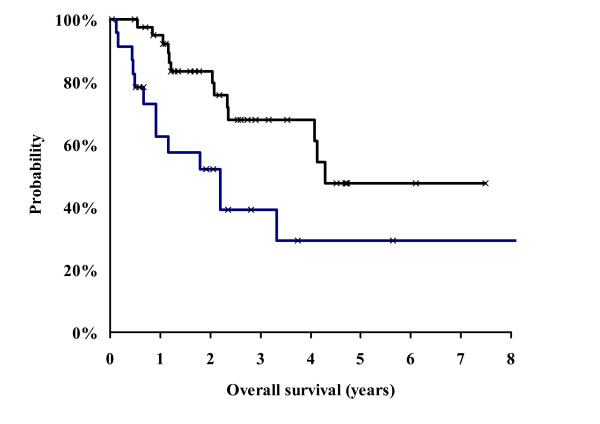
Kaplan Meier Analysis of survival probability. Comparison between patients who completed the SCHARC-scheme (n = 40, black line) and patients with modified therapy (n = 24, blue line). The Cox regression analysis showed a statistically significant difference between the groups (p = 0.01).

The three years overall survival rate was 57 % for all patients (intention to treat analysis, Fig. 1) and 68 % for those patients who were treated without protocol violation (Fig. 2). The patients with protocol modifications showed a significantly (p = 0.02) worse outcome compared to those without modifications. The three years overall survival rate for these patients was only 39 % (Fig. 2).

**Figure 3:**
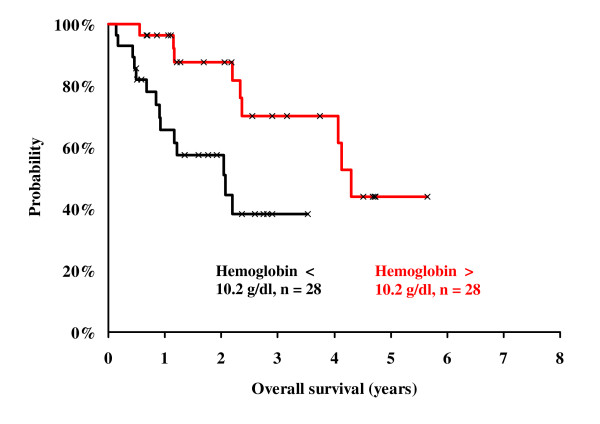
Kaplan Meier analysis of survival probability. Comparison between patients with hemoglobin nadir above 10.2 g/dl (n = 28, red line) and patients with a hemoglobin nadir below 10.2 g/dl (n = 28, black line). The Cox regression analysis showed a statistically significant difference between the groups (p < 0.01).

Stratified by means of the pre-therapy hemoglobin value (cut off: 12.5 g/dl (median)) we found no influence of hemoglobin on survival probability (p = 0.97). Stratified using the hemoglobin nadir (cut off: 10.2 g/dl (median)) we found a significant influence of hemoglobin on survival probability (3 years OSR of 70 % vs. 38 %, p < 0.01, Fig. 3).

Locoregional recurrences occurred in 15 patients and 6 patients developed distant metastases. The estimated probability of freedom from local treatment failure (FLTF) and the metastases free survival rate were 60 % and 84 % at three years, respectively.

### Acute toxicity

Grade III skin toxicity with intensive erythema and confluent moist desquamation was found in 53 % of patients and in 47 % grade II toxicity was observed (Table 2). During therapy most patients suffered from intensive mucositis. Mucositis was staged as grade II and III in 50 % and 42 % of the patients, respectively. 5 % of the patients developed a grade I mucositis. In 3 % of patients no grading of mucositis was performed (Table 2). Acute grade II – III dysphagia was very frequent (76 %). Mild or no dysphagia (grade 0-I) was exceptional (8.5 %). In 8.5 % of patients no data on the grade of dysphagia were available. 20 % of the patients developed pathological creatinin values during therapy (CTC grade 1–2), 78 % had no pathological creatinin values during therapy and in one patient (2 %) creatinin data were not available. Hematological toxicity was relatively low (Table 2). Only 1 patient had a hemoglobin level below 8 g/dl and 7 patients had leukocyte counts of >2 – 1 × 10^9^/l (CTC grade III) during therapy. No grade IV toxicity was observed in any of the 64 patients. After completion of therapy all patients were asked how they had tolerated therapy. One patient evaluated the tolerance as very good, 24 patients stated that they tolerated the therapy well (43 %), 24 (43 %) considered tolerance as moderate and 7 (13 %) patients stated that they tolerated therapy badly (n = 2) or very badly (n = 5).

### Late toxicity

The leading symptoms of late radiation toxicity were dryness of mouth and changes in taste. 92 % of patients reported a moderate to complete dryness of mouth. Slightly (grade I – II) and markedly (grade III) altered taste was observed in 50 % and 7 % of the patients, respectively. 43 % of the patients had no change of taste. Grade I and II hoarseness was found in 28 % and 5 % of patients, respectively. 67 % of the patients had no problems with speech in the course of the follow up time.

## Discussion and Conclusion

The presented analysis revealed fairly good survival data obtained from patients treated with the SCHARC scheme and an influence of hemoglobin nadir on prognosis.

### Outcome and toxicities, the Regensburg data versus a previously published phase II study

Recently, Ernst-Stecken et al. published the results of a phase II study encompassing patients treated from 1991 to 2002 with a comparable scheme of split course hyperfractionated radio-chemotherapy with 5-FU and DDP [7]. Because this and our study used a very similar treatment schedule, it is important to compare the study results of both investigations. We achieved a three year overall survival rate of 57 % compared to 53 % in the study of Ernst-Stecken et al. [7]. The local recurrence and metastasis free survival rates were also similar to our study with 60 % and 84 % versus 77 % and 73 %. The skin toxicity and the degree of mucositis were more intense than in the reference study [7]. We estimated 58 % of the patients skin reactions and 42 % of mucositis as grade III whereas the corresponding values of the reference study were 18 % and 13 %, respectively [7]. This discrepancy is explained by the tendency to overestimate the grade of side effects in clinical routine and the more critical approach how a patients sequelae should be scored within a study. Another reason could be that supportive care was different between the institutions. Interestingly, the patients estimated their therapy tolerance as good or moderate in 86 % of cases and only in 14 % as bad or very bad.

In spite of notable toxicity we were able to reproduce the remarkable survival data of Ernst-Stecken et al. which were also comparable to other studies assessing curative treatment in advanced head and neck cancer [2,3,7,18,19].

### Hemoglobin nadir as a prognostic factor

Anemia is a major cause of tumor hypoxia influencing not only radiosensitivity of tumor cells but also triggering irreversible changes in tumor biology [8,20-26]. A strong correlation of polarographically measured pO_2 _to uPA-levels in tissue of head and neck cancer was described recently [26]. The enzyme uPA is a VEGF-dependent factor involved in the degradation of the extracellular matrix and neovascularization. It has also been described as a predictor for metastatic spread and aggressiveness in breast cancer [26-28]. VEGF itself not only stimulates vascular proliferation and angiogenesis it also plays a key role in tumor invasion and metastatic tumor spread [29-31]. Dunst et al. found that hemoglobin values below 11 g/dl are predictive for upregulation of VEGF-levels in cancer patients as well as in patients without cancer [32]. Moreover, Becker et al. described a significant influence of severe anemia (below 11 g/dl) on tumor oxygenation [8].

However, we found no prognostic significance of pre-treatment hemoglobin values (p = 0.97). This finding contrasts the results of other authors and our previously published data [12]. The lack of prognostic significance of pre-treatment hemoglobin concentration might partly be explained by the fact that only 5 % of our patients had initial hemoglobin values below 11 g/dl which is considered to be the threshold for severe anemia in head and neck cancer. In our previous study in 1998, the percentage of patients with hemoglobin values below 11 g/dl was higher [12] (approx. 11 %, unpublished data). However, the hemoglobin concentration fell below the threshold of 11 g/dl [8] during therapy and reached a median value of 10.2 g/dl (nadir). Patients developing hemoglobin values below 10.2 g/dl in the course of therapy progressed significantly worse than those with higher hemoglobin concentrations (p = 0.01). This finding indicates that severe anemia during oncologic therapy influences the survival rate of head and neck cancer patients. The biological consequences of anemia therefore depend more on the hemoglobin level than on the time of measurement. Moreover, a correction of low hemoglobin levels could not revert the biological changes induced by anemia. This might be one reason why transfusions and erythropoietin applications were less effective as expected [15]. Therefore, severe anemia should be avoided during the complete course of therapy.

### Implications of Split course Schemes

Overall treatment time is known to be a predictor of outcome in head and neck cancer [15]. Therefore, it is strongly recommended to avoid intermissions during radio- or radiochemotherapy. Split course radiotherapy in standard fractionation schemes is known to be detrimental for head and neck cancer patients. However, the disadvantage of a pause can be compensated by accelerated hyperfractionation [3]. In this case, overall treatment time is not prolonged compared to normofractionated schedules. In a recent study, patients representing a selected group with unfavorable risk factors such as bad general condition, concurrent diseases or advanced tumors were treated with a 2 × 2.1 Gy split course schedule and Carboplatin [33]. Despite high daily doses this treatment scheme was considered feasible and the unfavorable survival rates were accounted for negative selection [33]. In a further meta-analysis by Budach et al. no advantage but also no disadvantage was found for hypo-fractionated split course schemes in comparison to normofractionated treatment schemes with the same dose as long as the total treatment time was identical [3].

However, even though some aspects of intermissions are negative others might be positive. One the one side, we fear proliferation or repopulation of tumor cells during a split. It is estimated that this effect reduces the isoeffective dose up to 0.6 Gy per day [3,34,35]. On the other hand we hope for reoxygenation of the tumor to enhance radiosensibility. Some authors measured an increased pO_2 _level after a fortnight break but this is not a proof of reoxygenation [36-39].

The addition of chemotherapy can also help to compensate possible negative effects of split course regimens. Moreover, it is hardly feasible to apply such aggressive combination of radio- and chemotherapy without a planned or unplanned pause. This pause enables normal tissue to recouperate and furthermore reduces the acute non hematologic toxicity. Therefore, when combining accelerated hyperfractionation with "poly-chemotherapy" a defined pause at a defined dose can be justified. This is confirmed by the results of our and the study of Ernst-Stecken et al. [7]. The three years overall survival rates of both studies were above 50 % in spite of nearly 90 % of UICC stage IV patients.

Due to various reasons, 38 % of the patients planned for treatment according to the SCHARC protocol received a somewhat differing treatment. In contrast to prospective studies with clearly defined inclusion and exclusion or break off criteria, an increased willingness to modify our clinic specific treatment scheme to reduce therapy-associated morbidity existed. However, it is unclear if the observed worse prognosis in the modified treatment scheme was due to the change of therapy or an epiphenomenon caused by negative selection.

We therefore conclude that the SCHARC regimen seems highly effective in spite of a high rate of T4 (64 %) and stage IV patients. Despite promising data the significance of split course RCT in SCCHN is unclear. It therefore seems justifiable to analyse the benefit of this scheme in a randomised, prospective study. Moreover, we found an influence of hemoglobin nadir on the prognosis.

## Competing interests

The author(s) declare that they have no competing interests.

## Authors' contributions

PS initiated the analysis of the data, supervised data collection and statistic and drafted the manuscript. KP and TK collected the data and performed the statistical analysis. CS participated in the study design and contributed in interpretation of data. OK and LS helped to draft the manuscript and contributed substantially to the final version of the paper. All authors read and approved the final manuscript.

## Pre-publication history

The pre-publication history for this paper can be accessed here:



## Figures and Tables

**Table 1:** Demographic and tumor related parameters of the 64 patients.

**Patients' characteristics**		
***Parameter***	**No.**	**%**

*Age (years)*		
Median (mean ± SD)	55 (56 ± 8)	
Range	38 – 71	
		
*Gender*		
Male	55	
Female	9	
		
*Tumor site*		
Oral cavity	19	30
Nasopharynx	5	8
Oro-/Hypopharynx	34	53
Larynx	6	9
		
*T-category*		
T1	0	0
T2	6	9
T3	16	25
T4	41	64
Tx	1	2
		
N-category		
N0	7	11
N1	6	9
N2	35	54
N3	8	13
Nx	8	13
		
Grading		
G1	2	3
G2	44	69
G3	17	27
Gx	1	2
		
*UICC-stage*		
III	8	13
IV	56	87

**Table 2:** Acute toxicity according common toxicity criteria (CTC) classification ((Trotti et al., 2000).

**Intensity grade**	**0–1**	**2**	**3**	**4**	**no grade**
***Acute toxicity (CTC)***					

Mucositis	3 (5 %)	32 (50 %)	26 (41 %)	0 (0 %)	3 (5 %)
Skin (including erythema and epitheliolysis)	0 (0 %)	30 (47 %)	34 (53 %)	0 (0 %)	0 (0 %)
Anemia (nadir)	34 (53 %)	22 (34 %)	1 (2 %)	0 (0 %)	7 (11 %)
Leucopenia	32 (50 %)	16 (25 %)	7 (11 %)	0 (0 %)	9 (14 %)
